# The Bacteriocins Produced by Lactic Acid Bacteria and the Promising Applications in Promoting Gastrointestinal Health

**DOI:** 10.3390/foods13233887

**Published:** 2024-12-02

**Authors:** Mohamedelfatieh Ismael, Mingxin Huang, Qingping Zhong

**Affiliations:** Guangdong Provincial Key Laboratory of Food Quality and Safety, College of Food Science, South China Agricultural University, Guangzhou 510642, China; 2018071076@nwafu.edu.cn (M.I.); 18026282269@163.com (M.H.)

**Keywords:** lactic acid bacteria, bacteriocins, gastrointestinal health, application

## Abstract

Bacteriocins produced by lactic acid bacteria (LAB) are promising bioactive peptides. Intriguingly, bacteriocins have health benefits to the host and may be applied safely in the food industry as bio-preservatives or as therapeutic interventions preventing intestinal diseases. In recent years, finding a safe alternative approach to conventional treatments to promote gut health is a scientific hotspot. Therefore, this review aimed to give insight into the promising applications of LAB-bacteriocins in preventing intestinal diseases, such as colonic cancer, *Helicobacter pylori* infections, multidrug-resistant infection-associated colitis, viral gastroenteritis, inflammatory bowel disease, and obesity disorders. Moreover, we highlighted the recent research on bacteriocins promoting gastrointestinal health. The review also provided insights into the proposed mechanisms, challenges and opportunities, trends and prospects. In addition, a SWOT analysis was conducted on the potential applications. Based on properties, biosafety, and health functions of LAB-bacteriocins, we conclude that the future applications of LAB-bacteriocins are promising in promoting gastrointestinal health. Further in vivo trials are needed to confirm these potential effects of LAB-bacteriocins interventions.

## 1. Introduction

Lactic acid bacteria (LAB) are useful bacteria that produce different types of metabolic bioactive components, such as hydrogen peroxide (H_2_O_2_), organic acids, diacetyl, bacteriocins, etc., which have antimicrobial activity and could work as agents to prevent pathogens invasion [1,2]. LAB probiotics are the most popular producers of bacteriocins that have health beneficial characteristics [3]. Bacteriocins are defined as a group of antimicrobial bioactive peptides that are ribosomally synthesized, proteinous in nature, and extracellularly released; most are cationic, and can exhibit a narrow or broad range of inhibition effects against pathogens that cause several health problems [4]. Bacteriocins are classified into Class I, Class II, Class III, and Class IV [5]. These bioactive peptides are usually produced as secondary metabolites during the stationary phase of bacterial growth cycle. They are composed of 2−10 amino acids synthesized as biologically inactive peptides within the parent proteins with an N-terminal leader pre-peptide attached to the C-terminal pro-peptide. The N-terminal has many functions, such as working as a recognition site to mature and transport protein, keeping the bacteriocin inactive to protect the strain from harming itself and interacting with the pro-peptide domain to ensure it is in the correct conformation (by enzyme–substrate interaction) [6,7].

**Figure 1:**
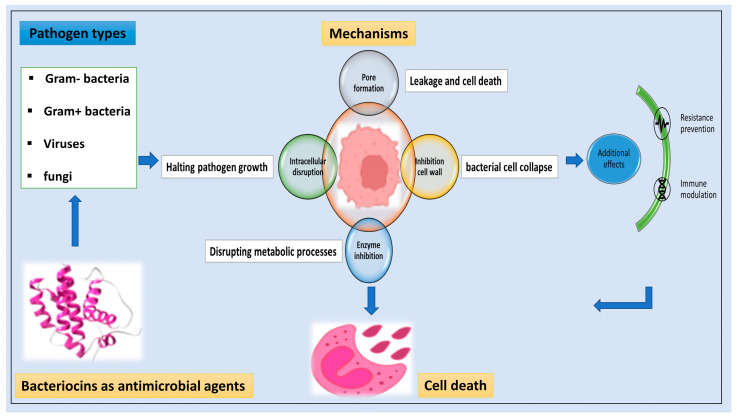
The general antimicrobial mechanisms of bacteriocins.

Additionally, LAB-bacteriocins have gained the interests of researchers due to their abilities as food bio-preservatives with inhibitory activity on several foodborne pathogens, or as promising therapeutic agents against clinical pathogens [8]. The general antimicrobial mechanisms of bacteriocins are shown in Figure 1. In addition, LAB-bacteriocins have distinguish characteristics such as permeation, good cellular diffusion, and low toxicity [9]. LAB-bacteriocins have several modes of action as potential intervention against gastrointestinal (GI) diseases, such as forming pores on the cell membrane to cause antagonistic killing, competing on adhesion sites inside the gut, inhibiting cell wall biosynthesis, inactivation of enzymes, disruption of proton motive force, and leakage of bio-molecules, leading to apoptosis [10]. Furthermore, their roles in promoting intestinal health include colonizing the intestine, modulating the gut microbiota, eliminating pathogens, and regulating the signals of the immune system. On the other hand, the physiological functions of LAB-bacteriocins include improving intestinal epithelial barrier to prevent pathogen invasion and tissue damage [11] and coordinating the mucosal barrier to maintain the health of the intestinal epithelial barrier, which acts as an efficient defense system for clearing pathogens [12].

Furthermore, LAB-bacteriocins can be applied in the food industry and medical field due to their unique traits, such as being odorless, tasteless, and colorless, with high remaining activity at thermal stress and acid environments [13]. Importantly, they can be easily degraded due to their proteinaceous nature and do not cause bacterial drug resistance [9]. Therefore, LAB-bacteriocins are potential alternative treatments to antibiotics due to their high activities even at low concentrations and their fast action against the targeted cells.

The gastrointestinal (GI) tract is one of the most important systems in the body. Its primary functions include gulping and digesting food, absorbing nutrients, secretion of enzymes, and excretion of water and waste (defecation). The GI system consists of various parts, namely the oral cavity, esophagus, pharynx, stomach, large intestine, small intestine, and anal canal, in order. In contrast, there are glandular organs working as accessories to GI, including the pancreas, liver, and gallbladder [14]. GI infections are one of the major common causes of mortality and morbidity worldwide. The health of this system can be affected by many factors depending on the diet and the environment; various diseases can infect it to cause intestinal disorders such as *Helicobacter pylori* infection, colonic bacterial infections like *Clostridium difficile*, *Escherichia coli*, *Shigella* spp., *Campylobacter jejuni*, *Salmonella typhi*, and *Yersinia enterocolitica*, colorectal cancer (CRC), diarrhea, virus diseases, IBD, and IBS [15].

This review aims to present an overview and a profound understanding of the functions of LAB-bacteriocins and their contribution to therapeutic applications in preventing and treating intestinal diseases. In addition, an insightful point of view was provided according to recent studies of potential uses of bacteriocins in promoting GI health and preventing diseases. To the best of our knowledge, this topic has not been addressed extensively in previous reviews and the use of strength, weakness, opportunities, and threat (SWOT) analysis has not been carried out to evaluate the potential health-beneficial effects of LAB-bacteriocins and their underlying mechanisms in infections control. This discussion will be helpful for the future applications of LAB-bacteriocins in GI interventions, inspiring novel strategies based on probiotics of LAB and their bacteriocins in the fields of biomedicine.

## 2. Current Treatments and Potential Intervention Effects of LAB-Bacteriocins on Gastrointestinal Diseases

### 2.1. Colonic Cancer

Cancer is the leading cause of death among non-communicable diseases in the world, despite the advancements of scientific research in finding suitable treatments and prevention strategies for various cancers [16]. Various factors and processes are associated with the emergence of cancer, such as aging, diets, lifestyle, oncogenic infections, genetics, and the variability of the gut microbiomes [17]. The characteristics of malignant cancer cells gradually develop across a series of mutations commencing by accumulating the normal cells. Over time their functions become dysregulated, thus, leading to a very severe, invasive, and proliferative disease. Eventually, these normal cells become mutant cells, which are heterogeneous, proliferative, have tumor clonality, and are more differentiated than normal cells [18]. Importantly, any tissue in the body could rapidly develop into cancer, specifically the tissues of breasts, lungs, skin, GI tract, and prostate. GI cancer is one of the most prevalent cancers such as colorectal cancer, which affects the colon and rectum; its major symptoms are bloody stool and reduced body weight of patients [19,20].

#### 2.1.1. Current Treatment Options for Cancer

The available treatments for GI cancer vary depending on the infected part and the stage of cancer, including three strategies, two of which are being explored in some cases [21]. The first one is surgical treatment: oncologic surgery is one of the cancer control and treatment strategies that can eradicate the early stages of cancerous tumors by inhibiting the spread and development of the carcinogenic cells in the body. Meanwhile, it increases patient survival rates, relieves symptoms, avoids recurrence, and gives the patients a better quality of life after the surgery. However, surgical treatment is an effective way to eradicate the early stages of cancer before the late spread stage among organs; surgical treatment often needs to be strengthened with further therapies such as chemotherapy to ensure that the cancer cells do not return again after they are removed [17]. The second is chemotherapy that leads to the reduction in size of the cancerous tissue in two ways—either by reducing the production of cancer cells or increasing the loss of cancer cells and their apoptosis. The problem of chemotherapy is its interference with cells and its cytotoxic effect on cell division, which leads to a decline in the production of new cells, which facilitates the invasion of other pathogens and develops resistance to cancer cells. The therapeutic failure of patients occurs due to the emergence of genetic changes in cancer cells caused by chemotherapy affecting the mechanisms of cell resistance; this is a huge challenge we could face. The third is radiotherapy, which is a treatment for controlling cancer cells in tumor areas by exposure to radioactive particles with high-energy doses to cause cells damage and cell death and reduce the risk of local recurrence and regional lymphatics [9]. Other strategies being investigated are targeted therapies by inhibitors of immune modulation [21]. However, the ideal cancer treatment is to completely remove tumors and metastases, but these treatments have some drawbacks. For instance, they either require additional treatments or lack target specificity to spare normal tissues, develop resistance in the cancer cells against therapies in the curing period, and additionally have several side effects [22]. Hence, significantly, there is an urgent need to find new treatment alternatives to counter this rapid growth of cancers.

#### 2.1.2. Bacteriocins as Potential Anticancer Agents

**Figure 2:**
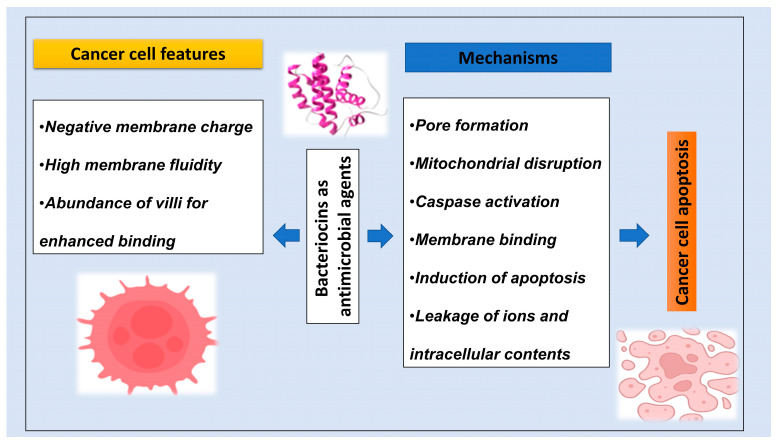
The proposed anticancer mechanisms of bacteriocins.

Bacteriocins are potential anticancer agents due to their mode of action that could inhibit the growth of cancer cells with less side effects and better precision in differentiating cancer and non-cancer cells [23]. The anticancer activities of probiotics and their bacteriocins have been explored by researchers recently by using in vitro and in vivo models to prove that bacteriocins are promising agents for targeting cancer cells [24,25]. Several bacteriocins studies have been conducted to identify the anticancer properties of bacteriocins, which confirmed that many LAB-produced antimicrobial peptides like bacteriocins have significant activity to kill or inhibit the growth of numerous cancer cell lines. The characteristic of cancer cells showed that their membrane surfaces have a negative charge, while the specificity of bacteriocin peptides to target cancer cells is mainly because they are cationic and amphiphilic and, thus, precisely bind to negatively charged membranes. Another feature is the membrane of cancer cells has a high fluidity, which destabilizes the cell membrane and has a complex flexibility attributed to existence of gangliosides, heparin sulfates, O-glycosylated mucins, and anionic phosphatidylserine, while non-cancer cells have neutral phospholipids [19]. Moreover, the membrane of cancer cells contains a large number of villi, which permits binding of a higher quantity of bacteriocin peptides; thus, bacteriocins selectively target the cancer cells and easily disrupt their membrane without affecting healthy cells [26]. This unique feature of binding and uptake of bacteriocins leads to lysis of the cell membrane, ultimately causing apoptosis of cancer cells. Figure 2 showed a schematic representation of the proposed anticancer mechanism of bacteriocins.

Several studies have proven that LAB metabolic products could inhibit the growth of cancer cells. Table 1 showed diverse of bacteriocins that exhibited anticancer activity with the proposed mode of action. Nisin is the most well-studied as an antibacterial agent, despite its reputation as a good agent, knowledge regarding its ability to inhibit cancer cells is still in the early stages. Norouzi, Salimi [27] demonstrated that Nisin could impair cells by perforation the cell’s membranes and forming pores; thus, those pores may allow calcium ions to enter the cells, which induces the apoptosis pathway by activating cell surface death. Ahmadi, Ghollasi [28] reported another mechanism wherein nisin could increase the apoptotic index of colorectal cancer cells (CRC) by promoting apoptosis signaling via the intrinsic pathway of mitochondria. It modulated the secretion of the B-cell lymphoma 2 (BCL-2) proteins family, such as pro-apoptotic BCL-2 proteins-associated X protein (BAX) and antiapoptotic BCL-2 proteins. Additionally, bacteriocins of enterocin-B, Pediocin K2a2-3, and Plantaricin P1053, which are produced by Enterococcus faecium por1 and Pediococcus acidilactici K2a2-3 isolated from the intestine of Philippine water buffalo, and Lactiplantibacillus plantarum PBS067 isolated from the feces of healthy humans, respectively, all exhibited anticancer activities against cell lines of HeLa, Caco-2, HT-29, and AGS [29,30]. Fluorescence microscopy imaging clearly showed morphological changes such as the formation of apoptotic bodies and membrane swelling. These results indicated that LAB-bacteriocins are promising anticancer candidates and could complement and potentially replace conventional cancer treatments. Clearly, the current research is mostly conducted in vitro with limited in vivo studies and clinical trials assessing long-term effects, bioavailability, and pharmacokinetics. Therefore, to translate these findings into actual treatments, the more in-depth in vivo investigations are required, such as safety assessment, efficacy, and biodistribution, as well as mining novel bacteriocins with good properties, developing delivery systems, enhancing bioavailability, and reducing systemic toxicity. Meanwhile, studies of dosing, immunogenicity, combination with exist treatments, scalability of production, and cost-effectiveness will bridge the gap between experimental research and clinical applications.

### 2.2. Helicobacter Pylori Infection

*H. pylori* is a Gram-negative bacterium commonly causing health problems, roughly 4.4 billion people are globally infected. The people at risk of its infection are mostly stomach ulcer patients; around 70% of cases have gastric ulcers. After *H. pylori* colonizes the gut, it produces many metabolites, like protease, urease, phospholipases, etc., as virulence traits; these secretions could affect the protective mucous coating of the stomach and duodenum and cause weakening and damage to the layer, which may lead to irritation of the lining and causes chronic dyspepsia, gastritis sore or ulcer, mucosa-associated lymphoid tissue lymphoma, and gastric adenocarcinoma [31].

#### 2.2.1. Current Treatment Options for Cancer *H. pylori* Infection

Peptic ulcers are usually treated with various antibiotics (such as tetracycline, amoxicillin, metronidazole, ampicillin, erythromycin, clarithromycin, and bismuth), proton pump inhibitors, antiacids, and H2 blockers to eradicate them. Currently, triple therapy consisting of amoxicillin, clarithromycin, and omeprazole is a widespread treatment to manage infection. Despite that, the developed countries are proposing quadruple therapy by omeprazole, tetracycline, bismuth sub-citrate potassium, omeprazole, and metronidazole as a principal treatment [32]. Recently, the emergence of antibiotic-resistant *H. pylori*, which carries point mutations, has raised global concern, and antibiotics binding to ribosomes has resulted in a reduction in eradication rates. In addition, current antibiotics have side effects such as nausea, vomiting, abdominal pain, and bloating; this leads to poor patient adherence to the prescribed medication and then encourages the emergence of MDR strains. Considering the above, finding an alternative treatment as a novel therapeutic approach for *H. pylori* infection is crucial. As a result, numerous probiotics and their metabolites are being extensively investigated.

#### 2.2.2. Bacteriocins as Potential *H. pylori* Infection Agents

LAB-derived bacteriocins have potential therapeutic applications in controlling or eradicating pathogens in the gastrointestinal tract such as *H. pylori*, which will provide new attractive alternative to get rid of conventional treatment approaches [33]. Laboratory studies have shown selective toxicity of bacteriocins against certain bacteria, and research has explored their potential as an alternative or supplemental treatment option for *H. pylori* infection. These compounds may offer advantages such as specificity against targeted bacteria and a lower propensity for inducing resistance compared to conventional antibiotics. Below, some examples of bacteriocins from various studies are listed in Table 2. A164 lacticin BH5 produced by *Lactococcus lactic* subsp. BH5, which was isolated from kimchi, Korean fermented vegetables, bulgaricin BB18 produced by *Lactobacillus bulgaricus* BB18, which was isolated from authentic Bulgarian dairy products, bacteriocins of *L. delbrueckii* subsp. *bulgaricus* (GLB) strains, NH2-7C produced by *Lactococcus* sp., and NH2-7C from Thai fermented pork (Nham) have adequately exhibited strong anti-*H. pylori* activity in in vitro experiments. Meanwhile, BK10, BK11, BK13, and BK61 produced by LAB isolated from Baikkimchi, as well as DCE 471 and La1 extracts of *L. amylovorus* DCE 471 and *L. johnsonii* La1 have shown the same activity [34,35,36,37]. In vitro studies indicated that the actions mechanisms of bacteriocins in combating *H. pylori* infection were destroying the cell cytomembrane structure and leading to the leakage of intracellular contents, while in vivo studies showed that bacteriocins significantly reduced the adhesion of *H. pylori* to human stomach adenocarcinoma cells and urease activity [33,38,39]. Thus, LAB-bacteriocin producers are good candidates to be used as starter cultures to manufacture functional products with potential health benefits, which might be associated with gut microbiota enrichment and promoting the abundance of beneficial bacteria. However, further research is needed to evaluate their effectiveness, safety, and clinical applicability in treating *H. pylori*-related diseases.

### 2.3. Multidrug Resistant Bacterial Infections

Infections associated with microbial drug-resistant (MDR) bacteria are rapidly spreading in environmental settings, reducing the effectiveness of antibiotics, and some bacteria have currently become resistant to almost all available antibiotics [40]. Therefore, these bacteria represent a public health problem and a source of worldwide concern due to the wake of rapidly growing antibiotic resistance escorted by the slower development of new antibiotics. In this decade, combating infections associated with MDR microbes is a major challenge and a threat to the existence of humankind, significantly increasing the global mortality rate; estimated by the UN, it will reach as many as 10,000,000 deaths by 2050 [5]. To tackle this situation, the World Health Organization (WHO) has urged the development of sustainable alternatives regarding antibiotic resistance. According to recent WHO data, there is an arsenal of 50 new antimicrobial agents, and their compositions are now under clinical development. Those compounds are (thirty-two) active antibiotics agents, (ten) biological origin agents, (two) active against MDR Gram-negative pathogens, and (two) innovative agents (World Health Organization, 2019a) [41].

#### 2.3.1. Current Treatment Options for Multidrug-Resistant Bacterial Infections

The conventional treatments to effectively control MDR bacterial infections are all the different classes of antibiotics, including bactericidal or bacteriostatic agents. Particularly, the most common drugs include quinolones, macrolides, penicillins, cephalosporins, monobactams, tetracyclines, and glycopeptides. Recently, treatments of antibiotic combinations have been approved to prevent MDR (Gram-) bacteria which include a combination of b-lactam/b-lactamase inhibitors such as meropenem/vaborbactam, ceftazidime/avibactam, ceftolozane/tazobactam, and aminoglycosides (plazomicin) with tetracyclines (eravacycline) [42,43]. The antibiotics can be classified into cell wall inhibitors, nucleic acid inhibitors, and protein synthesis inhibitors, which kill bacteria or prevent their multiplication. Nonetheless, misuse and overuse of these treatments since their discovery have contributed to the increase and development of resistance by bacteria.

**Table 1:** Studies of LAB-bacteriocins alleviating colorectal cancer using different cancer cell lines.

Bacteriocin and Producing Bacteria	Type of Cell Line	Effects	References
Nisin- *L. lactis*	LS180, SW780, HT29, and Caco-2 colorectal cancer cells	Reduced cell proliferation of LS180 (IC50 = 80–400 IU/mL), SW48, HT29, and Caco-2 (IC50 = 350,800 IU/mL) and reduced the expression of molecular biomarkers of MMPs and CEA for detection of colon cancer metastasis	[27]
Nisin- *L. lactis*	The colon cancer cell line of SW480	4000, 3000, 2500, and 2000 μg/mL of nisin led to anti-proliferative impact and augmentation apoptotic index via intrinsic pathways and lead to cancerous cell death	[28]
Plantaricin P1053- *L. plantarum* PBS067	E705 colon cancer cells	Inhibitory effect of cell proliferation of nearly 30% at 10 ng/mL	[29]
Pediocin K2a2-3 *P. acidilactici* K2a2-3	HT-29 and DLD-1 cell lines	Reduced the proliferation of human colon adenocarcinoma cells in a dose-dependent manner. Undialysed bacteriocin fractions inhibited (55.0 ± 4.8%) and (53.7 ± 7.0%) of HT29 and HeLa cells, respectively	[30]
Microcins-*Klebsiella pneumoniae*	HT29 and SW620 colorectal adenocarcinoma cell lines	Decreased in cancer cell Viability HT29 cell (treatment with 60 µg/mL reduces growth up to 50%) SW620 cell (treatment with 60 µg/mL reduces growth up to 69%) Significant reduction in SW620 tumor size	[44]
Enterocin-A + B *E. faecium* por1	The cell lines of the Human colon (HT-29) and gastric ulcer cells (AGS)	The bacteriocin enterocin-B showed 38.42, 28.16, and 22.84% of cell growth inhibition against HeLa, HT-29 and AGS cancer cells, respectively. The inhibition of cancer cell growth was significantly increased to 73.83, 57.94 and 51.76% when the HeLa, HT-29, and AGS cells were treated with the combination of bacteriocins (enterocin-A + B).	[19]
Microcin E492-*Klebsiella pneumoniae* RYC492	Colorectal carcinoma cells	Induces necrosis (>20 μg/mL) or apoptosis (5–10 μg/mL), which is associated with activation of caspases, the loss of mitochondrial membrane potential, and the release of calcium ions from intracellular stores	[45]

**Table 2:** Bacteriocins of LAB have activity against *Helicobacter* infection.

Bacteriocins	Producing Bacteria	Target	Effect Strategies	References
Lacticin BH5	*L. lactic* subsp. BH5	*H. pylori* ATCC 43504	In vitro inhibitory activity at 40,960 AU/mL with MIC of 12.5 mg/L	[33]
Lacticins A164	*L. lactic* subsp. A164	*H. pylori* ATCC 43504	In vitro inhibitory activity at MIC of 12.5 mg/L, the majority of cells had coccoid form and extensively damaged surfaces, the viable cells decreased by 0.58 and 1.62 logs after 12 and 24 h, of treatment respectively.	[33]
Bulgaricin BB18	*L. bulgaricus* BB18	*H. pylori* HPP112, HPK78, and HPKK160	In vitro inhibitory activity with inhibition zones 11 ± 0.3 to 12 ± 0.4	[35]
GLB44 and GLB47	*L. delbrueckii* subsp. *bulgaricus* (GLB44 and GLB47)	clinical *H. pylori* strains, including multidrug antibiotic resistance	The inhibition activity is of >81% of the test strains with mean inhibitory zone diameters of >13 mm) was found with both CFSs3 and CFSs4	[36]
NH2-7C	*Lactococcus* sp. NH2-7C	*H. pylori* ATCC 43504T, 3875, and BK 364, isolated from patients with gastritis and gastric cancer	In vitro inhibitory activity with antimicrobial activities (AU/mL) of 3200 and 6400	[37]
PLNC8	*L. plantarum* PLNC8	*H. pylori* ZJC03	Act on the cell membrane and their enzymes of *H. pylori* by destroying the cytomembrane structure, leading to the leakage of cell contents and thereby achieving a bactericidal effect	[38]
BK11, BK10, and BK13	*L. brevis* BK11, *L. plantarum* BK10, and *L. acidophilus* BK13	*H. pylori* ATCC 43504	In vitro inhibitory activity with inhibition zones (mm) of 11.8, 10.2, and 13.0 and antimicrobial activities (AU/mL) of 512, 128, and 64. They are effective in inhibiting the adhesion of *H. pylori* to human stomach adenocarcinoma cells and their urease activity	[46]
BK61	*E. faecalis* BK61	*H. pylori* ATCC 43504	Significantly reduced the number of cells of pathogen found adhering to the monolayers of cultured human gastric adenocarcinoma epithelial cell line and reduced the urease activity (*p* < 0.05).	[39]
DCE 471 and La1	*L. amylovorus* DCE 471 and *L. johnsonii* La1	*H. pylori* ATCC 43504 and six clinical isolates of (strains Hp1-Hp6)	Reduced the viability with more than 2 log units within 4 h of incubation (*p* < 0.001)	[34]

#### 2.3.2. Bacteriocins as Alternative Antimicrobial Agents

Currently, various strategies have been studied to prevent and treat antibiotic-resistant bacterial infections and control their spread through investment in scientific research to develop new therapeutic agents [47]. In response, bacteriocins are active antimicrobial peptides (AMP) and could be an interesting solution against clinically susceptible and drug-resistant pathogens. The bacteriocins produced by LABs are thermos- and pH- tolerant, odorless, tasteless, and eventually degraded into harmless amino acids, while antibiotics could generate harmful metabolites [48]. In addition, their highly cationic nature with disulfide bonds and cysteine residues that structurally help to target the eukaryotic defensins, which increase their acceptability to be applied in promoting human health [49]. Some potential LAB-bacteriocins as alternative antimicrobial agents against multidrug-resistant bacteria are listed in Table 3. LAB-bacteriocins exhibited many mechanisms against multidrug-resistant bacteria; according to in vivo studies, they mediated pro-inflammatory cytokine secretion, regulated theTLR4 signaling pathway, destroyed the membrane permeability, reduced the bacterial load, and inhibited the cell division [48,50,51]. However, in vitro studies indicated that LAB-bacteriocins induced pore formation on cells, induced cell cycle arrest, destroyed internal cell components via enzymatic degradation with material leakage, induced loss of cytoplasmic contents, induced the collapse of cells, and eventually caused structural deformities [40,52,53]. Undoubtedly, successful results in the development of bacteriocin-based therapeutics will contribute to clinical research on treating infections caused by MDR-Gram (+ and −) bacteria [54]. Interestingly, bacteriocins can be engineered to attach anywhere on the cellular outer membrane since they are non-specific receptors, and they can easily be modified by bioengineering, such as bacteriocins (Class II); these techniques do not have large post-translational manipulations and it is possible to obtain variants according to the required needs [55]. Therefore, this technique represents a new path in bacteriocins research, which could lead to the development of new drugs with potential therapeutic applications as an alternative to common antibiotics. Meanwhile, there is an urgency to increase and advance the models that evaluate the efficacy and possible toxicity and their side effects, which are the key factors in determining their possibility as therapeutic agents against multidrug-resistant infections.

### 2.4. Viral Gastroenteritis

Extensively, many viruses are known to cause acute viral gastroenteritis. Overall, astrovirus, rotavirus, sapovirus, norovirus, and adenovirus to children under five years old are the most important ones. Particularly, the most common cause of acute viral gastroenteritis is rotavirus infection with over 450,000 deaths/year. The pathogenesis of this virus in patients is due to two types of proteins. NSP1 is an important protein that down-regulates the inflammatory cytokines associated with interferon production, and NSP4 affects relative cellular electrolyte balance (interior and exterior), leading to diarrhea [56]. The complex interior factors, such as gut microbiome dysbiosis and disruption of homeostasis, can significantly and adversely impact viral immunity and the defense mechanisms of the host. While the exterior factors, such as accelerated mobility of goods and traveling passengers between countries, climate change, demographic change, and rapid economic growth, are also important factors in the spread of infections. In parallel together, these factors have led to the current rise in viral infections [57].

#### 2.4.1. Current Treatment Options for Viral Infections

The current conventional treatments of viral gastroenteritis are mainly chemical drugs including gene therapy, which focus on the regulation of pro- and anti-inflammatory cytokine expression like pegylated interferon-α (IFN-α). Ribavirin is a well-known one, acting as an antiretroviral recombinant therapy and as multiple antagonists to tumor necrosis factor-alpha (TNF-α). Other approaches include managing inhibitors of DNA polymerase activity to preclude the virus from proliferating and using the oral rehydration solutions to compensate for the loss of body fluids and electrolytes [58]. At the same time, the WHO recommends Zinc supplements to improve oral rehydration. Since 1998, the first rotavirus vaccine has been licensed and until now is implemented worldwide effectively [59]. However, the viral genomes could easily mutate to become resistant to all these therapies and may be prone to cause treatment failure. In view of this, resistant viruses are raising global concern and attracting attention to the importance of finding suitable prevention strategies and effective treatments. Till now, relatively few antiviral drugs have been developed to control their rapid spread; consequently, the discovery of novel methods to protect from viral pathogen diseases should be accelerated to control their devastating effects [60].

#### 2.4.2. Bacteriocins as Potential Antiviral Agents

Recent studies have indicated that small antimicrobial peptides, known as bacteriocins, have antiviral activity against several viral infections, through their ability to modulate the immune response and enhance the mucosal systems of GI [57]. Table 4 presents some LAB-bacteriocins with anti-gastroenteritis virus activities. For instance, Enterocin CRL35, ST5Ha, and NKR-5-3C and Erwiniaocin NA4 exhibited antiviral activity against the herpes virus. According to these studies, bacteriocins have two modes of action to inhibit viral infections. The first mechanism involves exhibiting anti-exhibiting activity of the virus on the mucosal surface at the first step of infection before the virus enters human cells. For example, enterocin CRL35 of *E. faecium* showed an ability to disrupt an early stage in the HSV infection cycle by lowering the synthesis of glycoprotein gD [61]. In addition, a novel lead peptide of LabyA1 presented consistent and broad activity against HSV with profound synergistic effects in combination with antiviral drugs of acyclovir, tenofovir, saquinavir, saquinavir, enfuvirtide, and raltegravir [61]. Conversely in the second mechanism, bacteriocins work after the entry of virus to the host at the late steps of the viral replication cycle to reduce the viral cytopathic effects and block the synthesis of glycoproteins. Regarding that, a study by Serkedjieva, Danova [62] found that *L. delbrueckii* produced a bacteriocin that only affected the late stage of influenza virus infection by reducing the production of viral proteins in infected cells. Drider, Bendali [63] also reported that the metabolites of *Bifidobacterium adolescentis* and *Lacticaseibacillus casei* did not prevent viral entry but were associated with reduced expression of the viral toxin NSP4. However, the information provided evidence that administration of LAB strains producing bacteriocins and/or just bacteriocins have a potential activity to prevent or inhibit the viral infections. Extensive studies about the mode of action and the exact defense mechanisms are still needed before bacteriocins can be applied in therapeutics.

**Table 3:** Effects of bacteriocins of LAB against MDR infections.

Bacteriocins	Producing Bacteria	Target Strain	Effect Strategies	References
Plantaricin bio-LP1	*Lactiplantibacillus plantarum* NWAFU-BIO-BS29	MDR-*Escherichia coli*	Partially ameliorated MDR-*E. coli* infection by reducing the inflammatory response through inhibiting the pro-inflammatory cytokines and strongly regulated the TLR4 signaling pathway.	[48]
Lactocin XN8-A	*L. coryniformis* XN8	MDR-*Listeria monocytogenes*, *Staphylococcus aureus* 2, *E. sakazakii* 14–18 (2), *Salmonella* 36T	LXA destroyed membrane permeability in vitro and induced pore formation of target cells. Furthermore, LXA induced cell cycle arrest at both G1 and G2/M phases by cell cycle analysis.	[52]
Thuricin CD	*Bacillus thuringiensis*	*Clostridium difficile*-associated disease-MDR *Clostridium difficile*	Inhibits non-host pathogenic strains from forming cell walls and destroys internal cell components such as DNA and RNA via their high proteolytic enzyme degradability.	[7]
Pediocin M31L	*P. acidilactici* LMG 2351	*L. monocytogenes* 10403S	Anti-listeria in a simulated human gut environment with negligible activity against a range of obligate anaerobic commensal gut bacterial species.	[64]
Lantibiotic lactin 3147	*L. lactis* DPC 3251	MDR- *S aureus*	Reduced the pathogen numbers in the liver, spleen, and kidneys of infected mice.	[65]
Nisin A Lacticin Q	Commercial Nisin *L. lactis QU 5*	Methicillin-resistant *S. aureus* MR23	Pore formation leading to ATP efflux, which is important for the bactericidal activity against both planktonic cells and biofilm cells.	[66]
Bacteriocin CH3	*L. lactis* CH3	MDR-*S. pyogenes*, *S. aureus*, *K. pneumoniae*, and *S. flexneri*	Disruption in membrane integrity of target cells occurred with intracellular material leakage, loss of cytoplasmic contents, collapse of cells, and eventually caused structural deformities.	[40]
Bacteriocin JM01	*P. acidilactici*	Methicillin-resistant *S. aureus*	Reduced the metabolic activity of cells in the biofilm formation of MRSA by inhibiting its growth and adhesion to a polystyrene surface.	[67]
Curacin A Pediocin A	*P. inopinatus* K35	MDR-*Pseudomonas aeruginosa*	Cell surface damage and shortened cell length were observed in the treatment group compared to the control group. At the end, this led to general deterioration in cells.	[68]
Lantibiotic mersacidin	*Bacillus* sp. *strain* HIL Y-85.54728	Methicillin-resistant *S aureus* (MRSA)	Mersacidin therapy revealed anti-MRSA activity in a serum bactericidal test, and no bacteria was detected in blood, lungs, liver, kidney, spleen, or nasal scrapings.	[69]
Lantibiotic NAI-107	Strains belonging to *Firmicutes* and *Actinobacteria*	Methicillin-resistant *S aureus* glycopeptide-intermediate *S. aureus*, and vancomycin-resistant *Enterococci*	Reduces the bacterial load in heart vegetations in a dose-proportional manner.	[50]
Penisin	A3 strain, genus *Paenibacillus*	Methicillin-resistant *S. aureus*	TEM result showed the shrinkage of cells upon treatment, most probably as a result of dehydration due to a compromised cell membrane. It is also an inhibitor of cell division.	[53]
Enterocin DD14	*E. faecalis* 14	Methicillin-resistant *S. aureus* (MRSA-1)	Showed better body weight recovery after the infection, and less pronounced histopathological alterations were observed, protecting colonic, liver, and spleen soft tissues and controlling the MRSA infection	[51]

### 2.5. Inflammatory Bowel Disease

Inflammatory bowel disease (IBD) is a nonspecific complex chronic inflammatory disorder in GI. Furthermore, IBDs comprise ulcerative colitis (UC) and Crohn’s disease (CD), which are long-term erythrogenic diseases. The infection rates of IBD are rapidly increasing in industrialized countries; research has shown that the etiology and pathophysiology are completely understood and suggest that different factors might aggravate it, such as diet types, psychological depression, and imbalance of microbiota (dysbiosis) [70]. Meanwhile, some intestinal microorganisms such as *Campylobacter concisus*, *E. coli*, and *Mycobacterium avium* are associated with IBD due to their excessive inflammatory response (foodborne diseases like diarrhea). Furthermore, some crucial factors play important roles in triggering IBD, like high oxidative and intestinal barrier dysfunction, which are involved in generation of high levels of reactive oxygen species (ROS) and induce pro-inflammatory cytokines secretion such as interleukins (IL-6 and IL-1β) and tumor necrosis factor (TNF)-α, respectively. A previous clinical study revealed that the expression of intestinal tight junction proteins (TJs) was significantly reduced in the tissues of IBD patients, which is particularly related to promoting intestinal permeability, while the excessive secretion of pro-inflammatory cytokines, including (TNF)-α, contributes to infection pathogenesis [71]. Additionally, other etiologies may have genetic susceptibilities, hereditary inclination, ecological triggers, and changes in the immune system. The apparent symptoms of IBD include abdominal pain, diarrhea, bloody stools, and weight loss. To investigate the IBD, many models such as dextran sulfate sodium-induced colitis have been used [49].

#### 2.5.1. Current Treatment Options for IBD

Many conventional drugs are used to treat patients with IBD. The current available drugs include sulfasalazine (SASP), anti-TNF-α antibodies, corticosteroids, and 5-aminosalicylic acid. However, all these treatments still have limited therapeutic efficacy, frequent harmful effects, and cannot completely cure the inflammatory infection due to its unclear etiopathogenesis and complex diagnosis [12]. Therefore, there is an urgent need to develop new bioactive drugs derived from natural resources, which are safe and without side effects for controlling long-term IBD inflammation.

**Table 4:** Antivirus effects of LAB-bacteriocins.

Bacteriocins	Producing Bacteria	Target Virus	Effects	References
Enterocin CRL35	*E. faecium* CRL35	HSV type 1 and 2	Lowering late glycoprotein synthesis. Seemingly it disrupting a slightly earlier step in the infectious cycle of HSV.	[61]
Subtilosin A	*Bacillus amyloliquefaciens*	HSV type 1 and 2	Disrupting late infectious stages of both HSV type 1 and HSV type 2.	[72]
Enterocins-like bacteriocin ST5Ha	*E. faecium* ST5Ha	HSV	Anti-HSV activity with a selectivity index (CC50/EC50) of 173.	[73]
GEn17	*E. durans*	HSV-1	Antiviral activity of for GEn17 before and after virus adsorption were recorded against HSV-1 39.2% and 71.6%, respectively.	[74]
Enterocins AAR-71 and ST4V	*E. faecium* AAR-71 *E. mundtii* ST4V	HSV type 1 and 2	Possess demonstrable antiphage activity witnessed as a reduction in PFU after treatment to a zero level.	[61]
Enterocin NKR-5-3C	*E. faecium* NKR-5-3C	HSV type 1	Showed antagonistic activity against HSV type 1 with CC_50_ lower than 1200 lg/mL, while the EC_50_ value was 30 lg/mL.	[75]
LabyA1	*E. durans*, *Geo9*, *Ge12*, and *He17*	HSV type 1 and 2	LabyA1 exhibited anti-HSV activity of (EC50s: 0.29–2.8 µM) against cell cultures as an entry inhibitor.	[76]

#### 2.5.2. Bacteriocins as Potential Anti-IBD Agents

Research on alternative interventions to IBD is still ongoing. While no conclusive evidence supports the direct roles of bacteriocins in managing IBD. Many studies suggested bacteriocins might treat colitis and alleviate the symptoms associated with IBD, such as halting bloody stools and diarrhea, decreasing weight loss and histopathological damages, improving colon length and intestinal permeability, as well as reducing the destruction of mucus layers, and exhaustion of goblet cells [49,71]. In vivo studies using animal models have shown promising results in managing inflammatory processes associated with IBD and reducing inflammation and have suggested that bacteriocins might impact microbiota composition, which supported their indirect influences on IBD and could have relevant effects in pathogenesis [49]. Bacteriocins studied for their potential effects on gut microbiota and inflammation, which might have implications for conditions like IBD. SDLKHFPF and SDIKHFPF, have shown promising results in attenuating colonic inflammation through protecting the intestinal barrier function and have promoted TJ protein expression of (occluding, occludens-1, and zonula) in the colon tissues, as well as suppression of myeloperoxidase and pro-inflammatory cytokines expression [49]. Five LAB-bacteriocin producer strains were isolated from the milk of breastfeeding mothers aged 21 to 45 years old and showed alleviation effects of IBD on 5- to 6-week-old BALB/c mice [71]. Results showed that these bacteriocins can alleviate the pathological symptoms of colitis in mice. The effect of Plantaricin EF (PlnEF) from wild-type *L. plantarum* NCIMB8826 on inflammatory responses in 2,4,6-trinitrobenzene sulfonic acid (TNBS)-induced mouse IBD was investigated [77]. *L. plantarum* D13 isolated from fermented food producing Plantaricin E, F, J, and K helps mice in their biomodulation of the gut microbiota after dextran sulfate sodium (DSS)-induced colitis [78]. Nisin A and Z obtained from culture of *L. lactis* subsp. *lactis biovar* UL719 isolated from raw milk cheese have shown the capacity to inhibit *C. difficile* cells and spore germination-associated diarrhea and pseudomembranous colitis [79]. In another study by Wang, Wang [12], a pectin/zein beads delivery system was used to investigate the effects of *Companilactobacillus crustorum* MN047-derived bacteriocin (CCDB) on colitis. On the other side, some bacteriocins were reported to have activity to kill or inhibit the intestinal pathogens (especially Gram-negative bacteria) that can cause IBD (see Table 5), such as bacteriocin of *E. faecium* KH24, which conspicuously affects the bacterial structure of mice feces by significantly increasing the abundance of *Lactobacillus* and reducing the number of *E. coli* [80]. A study on nisin of *L. lactis* and *Streptococcus lactis* showed that it can curb the growth and bacteria multiplication and their spores, especially Gram-positive like *Streptococcus hemolyticus* and *S. aureus*. Moreover, the secondary metabolite called reuterin secreted by *L. reuteri* has exhibited antibacterial activity with a broad spectrum against pathogens [81]. Furthermore, mice can be protected perfectly from *L. monocytogenes* infection by administration of Abp118 bacteriocin (class II) secreted from *L. salivarius* UCC118. In addition, EntV bacteriocin of *E. faecium* could inhibit hyphae and biofilm formation of *Candida albicans* and reduce the fungus virulence [6]. Rani and Tiwari [10] reported that Nisin effectively prevented the infection caused by *S. aureus*, and bacteriocins of lactocin AL-705, enterocin CRL 35, and pediocin PA-1 showed inhibitory activities against the growth of *L. monocytogenes* and prohibited their passage through the intestinal barrier. However, research exploring the potential health benefits of bacteriocins with further clinical investigations is needed.

The use of bacteriocins as a targeted therapy for IBD is still in its early stages. Although these studies provide insight into the potential mechanisms, their specific role, safety, and efficacy in managing IBD in humans require further investigation through well-designed clinical trials to investigate the direct effects.

### 2.6. Obesity Disorders

Obesity is a complex syndrome associated with a life-threatening metabolic disorder and its related complications predispose individuals to other diseases that may adversely affect human health [70]. As the consequence of a sedentary lifestyle and the high caloric food intake, obesity complications lead to overweight and accumulation of adipose tissue in various parts of the body till body mass index (BMI) exceeds 30 kg/m^2^. According to the World Health Organization, one-third of the world’s population is suffering from the influence of obesity, which is growing to be a global epidemic, and nearly four million people a year lose their lives because of obesity [82]. Studies revealed that the most common causes of obesity incidence have a positive correlation with gut dysbiosis and low-grade inflammations of some-morbidities such as cardiovascular, diabetes, colorectal cancer, and non-alcoholic fatty liver disease [83].

#### 2.6.1. Current Treatment Options

The current available strategies for the obesity management encompass the dietary management to reduce the direct intake of net energy, pharmacotherapy that is limitedly used due to unacceptable side effects and safety concerns, and surgical strategies [84]. Emerging research suggest that obesity can be controlled through various mechanisms and pathways of metabolic conditions by modulating gut microbiota which may play a critical role in weight regulation and metabolic health [85].

#### 2.6.2. Bacteriocins as Potential Anti-Obesity Agents

Bacteriocins have the potentiality to be anti-obesity agents, through regulating the signaling pathways of obesity disorders or re-modulating gut-microbiota, favorably influencing the balance of beneficial bacteria and indirectly affecting obesity-related parameters [86]. Several studies were conducted in vivo or in vitro to explore the potential influences of certain bacteriocins on gut-microbiota composition and metabolic health as shown in Table 6, indirectly affecting obesity-related parameters. For instance, commercial bacteriocin of nisin, gassericin A, six bacteriocins from human gut microbiota, PJ4 of *Lactobacillus helveticus* PJ4 isolated from rat feces, TSU4 from the fish gut, Plantaricin EFI of *L. plantarum* NCIMB8826-R, and garvicin ML (GarML) produced by *Lactococcus garvieae* DCC43 isolated from Mallard-duck gut, all these LAB-bacteriocins have influenced gut-microbiota composition significantly, and improved the metabolic health parameters, which lead to reduction in weight gain [83,85,87,88]. These promising findings have presented potential avenues for anti-obesity treatment, which could provide insights for future research. Further research to better understand their role, efficacy, and safety as a potential approach for directly managing or preventing obesity remains a subject of ongoing research.

## 3. The Potential Mechanisms of LAB-Bacteriocins Enhancing Intestinal Health

### 3.1. Gut Microbiota Enrichment and Regulation

The gut microbiota is a heterogeneous microbial ecosystem that constantly interacts with the host. Furthermore, interactions between the gut microbiota and the immune system of the host are essential for regulating colonic inflammation and maintaining homeostasis [11,90,91]. Conversely, an imbalance of gut microbiota known as dysbiosis is a crucial cause of colonic inflammation, which might lead to the occurrence of gut mucosal barrier damage and might exacerbate the loss of bacterial diversity during the infection and the overgrowth of pathogens [10].

Indeed, although LAB-bacteriocins show a relative narrow spectrum against pathogens, they can modulate gut microbiota; this property contributes to potential precision therapy for gut disorders [3,92]. Moreover, they have been proven in vitro to maintain cell homeostasis and enhance or repair the intestinal epithelial barrier, regulate the host physiological functions, and specifically boost immune responses. Also, bacteriocins interact with some receptors as signal peptides, which leads to reduced oxidative stress and increases the production of mucus by goblet cells, as well as enhancing the expression of epithelial tight junction proteins [93]. Therefore, bacteriocins may serve as significant effective therapeutic agents, which specifically eliminate intestinal colonization by pathogenic bacteria associated with intestinal diseases. They are nontoxic by selectively targeting harmful bacteria and potentially restoring microbial balance without profound disruption of the commensal gut microbiota [94]. Studies on gut microbiota modulation in animal models or in vitro experiments using bacteriocins or colonized peptides of certain intestinal microorganisms have shown promising results in altering gut microbial composition and could promote them acquire a competitive advantage to occupy established niches in the GI. For example, some positive effects have been observed for bactofencin A produced by *Lactobacillus salivarius*, on improving the intestinal communities of the host, essentially on anaerobic bacteria of *Clostridium*, *Bacteroides,* and *Bifidibacterium* spp. [63]. Consistent with the findings reported, small antimicrobial peptides of microcin H47 and microcin M were discerned by the catecholate siderophore receptors, which enhanced their competitiveness with other gut microorganisms [95]. In contrast, injection of salivaricin P in porcine ileum has increased the expression levels of IL-8, CD41 1 and CD81 1 double-positive cells [10]. Also, a single injection of Nisin F (640 AU) with lysozyme and lactoferrin in a mice model showed sufficient antimicrobial activity to kill or inhibit the gut pathogen population (e.g., *Listeria* spp. and *S. aureus*) [96]. A previous review of Donia and Fischbach [97] implicated that bacteriocins of ruminococcin A from *Ruminococcus gnavus*, salivaricins from *Streptococcus salivarius*, and *Clostridium nexile* and *Companilactobacillus crustorum* MN047-derived bacteriocins have vigorous activity to inhibit the growth of closely related pathogens and enriched the growth of beneficial microbiota. Similarly to the above, a two-component lantibiotic called (Cytolysin) produced by *E. faecium* has shown antibacterial activity against commensal human pathogens and prevents colonization [12,98]. In view of this, it seems that more research including clinical studies in humans are still needed to determine bacteriocins’ safety, efficacy, and therapeutic potential in managing the infections.

### 3.2. Promoting Short Chain Fatty Acids (SCFAs) Production

SCFAs are major secondary metabolic products of carbohydrate fermentation by LAB, such as acetic acid, isobutyric acid, propionic acid, butyric acid, valeric acid, and isovaleric acid [2]. These metabolic products have raised a growing interest due to their beneficial impacts on the human body, particularly, activating the innate immune system in the intestinal cells, which is the body’s first line of defense to mediate GI inflammation [48]. In addition, SCFAs, especially butyric acid, are known to increase the expression levels of TJs, which enhance the strengthening of the gut barrier function and decrease the inflammatory response by promoting innate immunity as well as regulating the expression of pro-inflammatory cytokines that alleviate colitis [99]. Meanwhile, SCFAs were proven to maintain the balance of the intestinal ecosystem and confer protection against colitis; therefore, the content of SCFAs can be considered as an indicator of changes in gut microbiota [12]. Studies by Ismael, Qayyum [48] and Pu, Hang [100] reported that LAB-bacteriocin administration exhibited a greater level of SCFAs and beneficial health effects.

### 3.3. Intestinal Epithelial Barrier

The intestinal epithelial barrier has an essential role in protecting intestinal tissues from out-invasion by preventing the entry of detrimental intestinal contents and pro-inflammatory stimuli into the lamina propria or even the circulatory system [101]. This epithelial barrier consists of tight junctions (TJs) (e.g., Zo-1, Claudins, and Occludins) and works as determinants of epithelial permeability [12]. Additionally, TJs have critical roles in intestinal tract health, where they organize the transport of nutrients in and out of the GI and block antigen entry to the circulation of the host. Indeed, occludin significantly maintain the adjacent cells Tj seal and also contribute to establishing contact with the actin cytoskeleton through the Zo-1 protein to constitute the plaque structures of TJs [102]. Meanwhile, mucins like MUC2 strengthen the tight junction structure, maintain the integrity and permeability of the intestinal epithelium, and prevent the damage of crypts and goblet cells. Conversely, damage the mucosa of the intestinal barrier increases intestinal permeability and could allow endotoxins and pathogens to enter the circulation and GI causing intestinal inflammation [48]. Evidently, IBD is directly significantly associated with damage to the intestinal barrier, like devasting the goblet cells and reducing their ability to produce mucus, which aggravates inflammation because of activating pro-inflammatory response and elevating of antigen translocation. The investigation results revealed that LAB-bacteriocins have high potentialities to protect the epithelial integrity of GI tract, and the main mechanism is covering the intestinal epithelium surface, which might lead to disintegration of enterotoxins and prevention of pathogen invasion. Bio-LP1 produced by *L. plantarum* and *C. crustorum*-derived bacteriocin (CCDB) had alleviated the intestinal barrier damage caused by multidrug-resistant bacterial infection and strengthened the gut barrier in DDS-associated colitis, respectively [12,48].

## 4. Challenges and Opportunities of Bacteriocins Applications

Currently, there is a critical need to discover new potent compounds to address the spread of lethal diseases due to limited capabilities of discovered drugs. Bacteriocins are potential alternatives, but there is still a need to improve their effectiveness and develop advanced methods to facilitate their mining, purification, characterization, and production steps [73]. On the other hand, bacteriocins that showed promising in vitro results should have an in vivo evaluation to assess their safety and interaction with the body before being authorized in medical applications. Furthermore, using bacteriocins in the medical field faces many challenges, such as the protease degradation problem or sensitivity of bacteriocins to protease; most bacteriocins are susceptible, being degraded by proteases, especially when administered orally, leading to the loss of antimicrobial activity. Many studies proposed various administration methods in vivo, such as feeding, intraperitoneal injection, and application to skin, or using delivery systems like nanoparticles (i.e., metal nanoparticles, nanospheres, organic nanoparticles, and nanofibers) or using probiotics and gels. However, to the best of our knowledge, those delivery systems have only limited effect in facing this challenge; therefore, selecting effective delivery methods is necessary to avoid bacteriocin degradation before reaching the intestine. Thus, LAB-bacteriocin-producing strains that colonize the gut can be used as delivery vehicles in studies of bacteriocin functions in the gastrointestinal tract. Moreover, future uses of bacteriocins can be upgraded through a deep understanding of the interactions between bacteriocins and nanomaterials [103]. A low yield of bacteriocins and weak antibacterial activity are also challenges, and using genetic engineering in LAB-bacteriocin producer strains can improve the productivity of bacteriocin and enhance the inhibition activity. Meanwhile, some bacteriocins are encoded by plasmids and are not produced in stable yields; therefore, these problems may be solved by genetic engineering, which is a promising solution [104].

On the other hand, many bacteriocins have great potential to be used as relatively narrow-spectrum antimicrobials for treating infection-related diseases in humans. Broad-spectrum antibiotic administration could significantly reduce microbiota diversity and cause gut imbalance. For example, the occurrence of diarrhea is associated with taking wide-spectrum antibiotics as side effects of the disorder of “good and bad bacteria” balance in the gut microbiota. Apparently, bacteriocins target specific bacteria compared to antibiotics with no impact on normal microbial flora; therefore, they have the opportunity to be used as an alternative treatment to antibiotics in the near future [105]. Moreover, bioengineered bacteriocins can provide alternatives to avoid the risk of rejection cases by the organism and reduce the associated side effects of cancer therapies. Another opportunity is using bacteriocins against multidrug resistant bacteria by understanding the mode of action that could help generate specific products used against MDRB. Furthermore, studies related to mining, purification, and identification of novel bacteriocins combined with existing peptides can tackle the increase in bacterial resistance to antibiotics. With the advancement of scientific technology, there has been an enormous increase in research on bacteriocins, especially on their unique functions, antimicrobial properties, and mechanism of action. These further studies will help advance their practicality in anticancer, anti-infection, and anti-inflammation or immunomodulatory applications, which makes them a potential alternative to conventional treatments [13].

Finally, using bacteriocins as alternative therapeutic agents to treat GI diseases and combat infections still needs urgently related research on their efficacy with conducting an advanced evaluation of their hemolytic activity, possible toxicity, side effects, distribution, and metabolism in the body. All of these are key factors to influence the successful usage of bacteriocins. In addition, it is necessary to conduct safety assessments on bacteriocins before applications, which include studying the potential toxin, immunogenicity and hemolytic effects, and evaluating the impacts on gut microbiota and long-term systemic exposure. Another aspect is the rapid emergence of drug resistance infections, which requires finding new alternatives to conventional approaches; therefore, bacteriocins could be considered to have advantages in filling this scientific gap [106].

**Figure 3:**
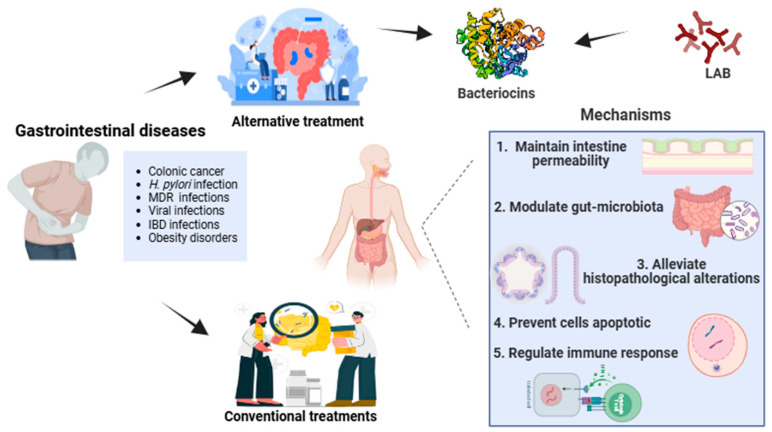
The mechanisms of bacteriocins alleviating GI diseases.

This review highlighted the potential applications of bacteriocins as alternative novel therapeutics to conventional treatments in gastrointestinal diseases and their promotion of intestine health (Figure 3). The opportunities for practical applications of bacteriocins are in the food industry as food preservatives and in the health filed as promising antimicrobial agents for GI disease, anticancer therapies, and microbiota modulation with more preclinical and clinical studies required. In the literature, there is an abundance of knowledge on the bacteriocins applied in food, while the available data on health-related applications are still limited. Consequently, this review provides a profound understanding of how bacteriocins will contribute to disease prevention and individualized therapies in future clinical practice and inspire novel strategies utilizing these products in the fields of biology and medicine. Nevertheless, the majority were only confined to in vitro experiments, and studies have not yet been adequate on in vivo models. In addition, there is no rigorous safety assessment of bacteriocins, which is very important before application and authentication. Among health applications, bacteriocins as antiviral agents have been far less studied and require further research to better understand their mechanisms.

## 5. Trends and Future Prospects

Conducting investigation studies on bacteriocins has been a new trend due to their promising future applications in different fields of industry and medicine. One of these aspects, bacteriocins, represent a potential drug alternative for replacing current medications to treat many intestinal diseases [107]. Consistent with the findings of progress in the antimicrobial peptides field, the conclusions were almost identical in bacteriocins retaining their inhibition properties in vivo and, simultaneously, showing a null or reduced toxic effect. According to the WHO, in 2019, 27 agents were in the stage of preclinical development as antibacterial peptides. The future prospects of LAB-bacteriocins not only lie in their discovery and identification but also in the need to determine their toxicity and safety to approve their safe use at clinical trials as therapeutic candidate agents [54]. Therapeutic proteins are rarely available in an oral dosage form due to the hostile environment with extreme pH in the stomach, the large size of the human gastrointestinal tract, the tight junctions of the intestinal epithelium, and immunological defenses in the intestinal mucosa, which, all together, are the barriers that make the oral delivery method difficult. This review discussed how bacteriocins penetrate the host’s body and how orally administered therapeutic proteins may be developed with similar mechanisms for delivery into the body. One of the promising aspects of solving this problem is using commensal bacterial components with the host to facilitate drug delivery. Here, patients may respond differently due to physical factors such as (ethnic, dietary, and geographic differences) or biological factors (such as the prevalence of bacterial strains among different populations, the behavior of the selected strains, and the inherited absence of particular bacterial strains in the gut). Another promising aspect is engineering the bacteriocins producer strains to control their proliferation by using therapeutic proteins tagged with bacterial peptides and identifying precise sequences that contribute to the evasion of host immunity. In this case, changes in the function and structure of therapeutic proteins induced by tagging should be evaluated, and additional mechanisms or guiding peptides may be required for selective delivery. Recent studies have focused on developing valuable strategies to prevent or treat intestinal diseases, such as considering the location of the activated enzymes when using bacterial molecular structures as the basis of prodrug conjugates to avoid the potential development of resistance arising from chronic exposure. Particularly, understanding the feature of leakage and limited entry across intestinal membranes is a way to find alternative methods to enhance the oral delivery of therapeutic agents and a way to discover how to reduce the intestinal membrane permeability to prevent the penetration of toxic bacterial materials. Nonetheless, some questions remain to be answered in how the body handles commensal bacterial materials and how they can escape from host surveillance before we can fully take advantage of this entry point to be considered as inspiring alternative methods to enhance the oral delivery of therapeutic agents with beneficial effects [108]. New insights may be considered in the future on using LAB-bacteriocins and investigating their beneficial influences against chronic and acute diseases like strep throat, asthma, the common cold, osteoporosis, Alzheimer’s disease, and psychiatric disorders.

Finally, it is very important to design and create new open access digital databases on internet websites, which contain information about identified bacteriocins related to their peptide sequences, classification, characteristics, mechanisms of action, and potential food and therapeutic applications. This will facilitate the work of researchers who can refer to the list of discovered bacteriocins and enable them to make comparisons to identify new bacteriocins in a much easier way. BACTIBASE, one of the most important integrated databases for obtaining information about bacteriocins, contains the largest amount of electronic information; however, it needs to be developed and continuously updated.

## 6. SWOT Analysis

SWOT analysis is a strategic planning technique that provides assessment tools based on identifying information internal sources (strengths of weaknesses) as well as external forces that may have uncontrollable impacts on decisions (opportunities and threats) regarding the use of the potential parameter [109]. This will lead to fact-based analysis, fresh perspectives, and new ideas, which can guide strategies that are more likely to be successful. Recent published research on bacteriocin applications in the medical field has shown that they have many opportunities to be applied in GI diseases because of the high demand in finding alternative treatment agents that can replace conventional interventions. However, various restrictions and barriers have hindered their direct utilization [64]. SWOT analysis might be a useful method to discuss the potential intervention effects of LAB-bacteriocins on GI health, which may eventually lead to the proposition of new strategies for bacteriocin development. Based on this information, we can make smarter decisions to make use of LAB-bacteriocins, capitalize on the strengths, mitigate risk regarding weaknesses, and plan for events that may adversely have an effect in the future.

The opportunities of using LAB-bacteriocins are promising. LAB-bacteriocins are innovative and excellent candidates for replacing traditional treatments. Many LAB strains have proven to be beneficial to the human body and frequently produce food-derived bioactive components like bacteriocins, which can be considered a potential therapeutic intervention for GI disorders and for improving intestinal health [110]. Additionally, bacteriocins could provide a good opportunity for widespread application in the medical field due to the growing problem of drug resistance.

The strength points lie in the possibility of using the rapid evolution of omic technologies to discover and identify novel bacteriocins with reduced cost. Moreover, bioengineering can improve their stability, activity, or specificity to target pathogens because the biosynthesis genes of bacteriocins are transferable, which are clustered on plasmids, transposons, and chromosomes [107]. At present, technologies of combination, conjugation, and fabrication of bacteriocins with other substances are emerging as future directions to achieve higher bacteriocin efficiency and develop their active packages [70].

The intrinsic weaknesses and shortcomings may limit the applications of LAB-bacteriocins. Some shortcomings should be addressed in the future, such as the production on a small-scale, the time-consuming process, the poor physical and chemical stability, and the loss or reduced activity due to their positive charge with a high content of hydrophobic macromolecules, which may be interacting with or binding to food ingredients. So, finding a delivery system for bacteriocins is critical for their potential applications in future therapeutics [111].

The most important threats to future applications of LAB-bacteriocins in human health are the stringent regulatory requirements for using new drugs. The currently available data on LAB-bacteriocins as alternative treatments seem to have a lack in evaluation reports of toxicity, long-term exposure, and safety evaluation. Moreover, the interaction between the immune system and bacteriocins has yet to be well investigated [7]. Consequently, all these reports are essential and pose a significant barrier to their widespread usage. Therefore, complementary clinical trials are crucially needed to make an informed decision on authorization and legislation regarding new drugs, which have become increasingly stringent.

**Figure 4:**
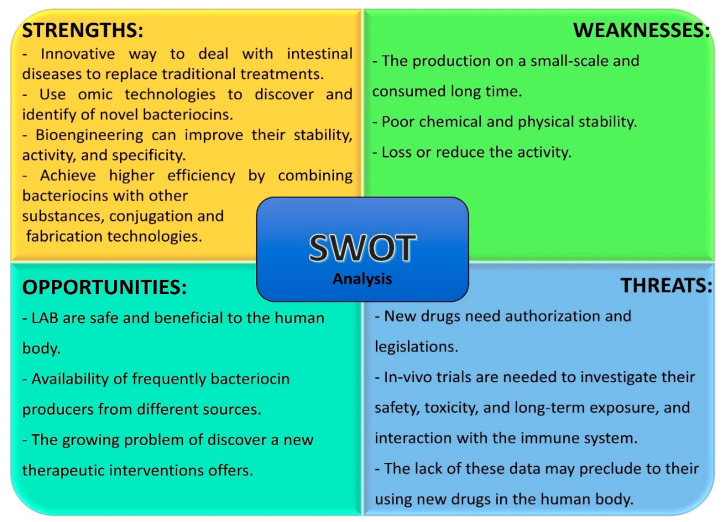
SWOT analysis of LAB-bacteriocins as potential therapeutic agents for GI health.

Figure 4 showed the internal factors on the top row and the external factors on the bottom row. In addition, the items on the left side are more positive/favorable aspects, while those on the right are more concerning/negative elements. Although all the points may not be of equal importance, all should represent key insights into the balance of opportunities and threats and advantages and disadvantages.

## 7. Conclusions

As discussed above, there is a growing need to find novel treatments to replace existing treatments for intestinal diseases, and they must be effective, especially with the increasing incidence of resistance to conventional treatments, the appearance of side effects or high inadequacy. Researchers have proven that bioactive components like bacteriocins are strong candidates for future medical applications as promising agents against multidrug-resistant bacteria, as an anti-IBD, as an anticancer, and as an antiviral therapeutic. Results in scientific studies have demonstrated that bacteriocins have shown high stability and distinctive properties that enable them to exert a variety of positive responses in the host’s body, such as modulating the immunogenic response after disease injuries, alleviating the adverse inflammatory effects in inflamed tissues, and reducing the biochemical parameters and histopathological damages associated with infection. However, most of these studies are in vivo, with limitations and scarcities in studies of their potentially in vivo effects. Consequently, more in-depth investigations in vivo and in clinical settings are urgent needed to assess their safety and clarify the properties of bacteriocins as therapeutic agents or promising substances for promoting GI health. In this regard, significant challenges arise for the use of bacteriocins and, therefore, there is a need to develop advanced strategies to face these challenges, such as low bacteriocins production that can be improved by co-culturing and statistical improvement techniques to increase yields and enhance bacteriocins’ antimicrobial spectrum. Increased attention and encouragement should be given to studies of bacteriocins using bioengineering and nanotechnology to increase their stability, expand their antimicrobial spectrum, as well as enhance the delivery to targeted cells. Further research in uncovering novel bacteriocins with good properties will help in gradually replace the conventional treatments by LAB-bacteriocins as a complement or alternative to current drugs to curb GI-diseases.

## Figures and Tables

**Table 5:** Bacteriocins of LAB have potential anti-colitis activity.

Bacteriocins	Producing Bacteria	Type of IBD	Effect Strategies	References
Peptide SDLKHFPF	*Tricholoma matsutake*	Dextran sulfate sodium (DSS)-induced colitis	Mitigated colitis by regulating TJ protein expression and pro-inflammatory cytokine production via NF-κB/MLCK/p-MLC signaling, improving the barrier function.	[49]
Plantaricin B, D, G, and EF	*L. casei*, *L. plantarum, L. rhamnosus*, and *L. acidophilus*	Acetic acid induced-UC	Disease activity index (DAI) scores, the weight loss, and the length of mice colon were improved in the treatment groups compared to IBD control group.	[71]
PlnEF	*L. plantarum* NCIMB8826	TNBS-induced mouse model of acute IBD	Exhibited intermediate levels of colonic tumor necrosis factor alpha (TNF-α) and interleukin-6 (IL-6) that ranged between the TNBS-treated animals and healthy controls.	[77]
PlnE, PlnF, PlnJ, and PlnK	*L. plantarum* D13	DSS-induced colitis	Decreased the disease activity index (DAI), altered the gut microbiota, reduced the weight loss, improved tissue regeneration, and reduced inflammatory cell infiltration.	[78]
Nisin A and Z	*L. lactis* subsp. *lactis biovar*	*C. difficile* associated diarrhea and pseudomembranous colitis	Inhibited the growth of all C. difficile isolates, with MICs 0.8 and 6.2 µg mL^−1^ for nisin A and Z, respectively, and the treated cells showed holes in the cell membrane and release of cytoplasmic contents causing cell death.	[79]
CCDB	*C. crustorum* MN047	DSS-induced colitis	Strengthened the gut barrier by increasing goblet cells and promoting the expressions of MUC2 and TJ-related proteins, inhibited the overexpressions of pro-inflammatory cytokines, chemokines, and pathogens/LPS-activated TLR4/NF-κB pathway.	[12]

**Table 6:** Reported bacteriocins of LAB with potential anti-obesity effects.

Bacteriocins	Producing Bacteria	Target	Effect Strategies	References
Nisin	*L. lactis*	Swiss NIH mice with fed a high-sugar diet	Lowered the body weights, abdominal fat, and body mass index, as well as significantly decreased the expression of *SCD-1*, *GLUT4*, 422(*ap2*), and *TNF-α*	[85]
Gassericin A	*Lactobacillus gasseri* LA39	3T3-L1 cells	Controlled the genes involved in adipogenesis, decreased expression of Stearoyl-CoA desaturase-1 gene in preadipocytes which reduced obesity related complications, enhanced production of Glucose transporter type 4 (GLUT4) in mice, which is associated with adiposity, and increased serum free fatty acid levels	[87]
Plantaricin S-beta, Carnolysin. Lactococcin B. Bacteriocin Iic, Plantaricin N, and Thermophilin A.	*Lactobacillaeacea* family	Molecular dynamic simulations analyses of the six top scoring SCD1 and bacteriocin complexes	Regulated the secretion of stearoyl-CoA desaturase 1 (SCD1) which is a key enzyme involved in the differentiation of adipocytes	[88]
PJ4 and TSU4	*L. brevis* PJ4 and *L. animalis* TSU4	Male C57BL/6 J mice with a high fat diet	Lowered weight of adipose tissue, controlled the production of IL-6, TNF-α, increased expression of PPARa and PPARg, and decreased resistin and adiponectin, and reducing obesity	[83]
Plantaricin EFI	*L. plantarum* NCMIB8826	C57BL/6J mice on a high-fat die	Overall 10% reduced the weight gain and also serum glucose levels in high fat diet mice which improved oral glucose tolerance	[86]
GarML	*L. garvieae* DCC43	BALB/C female mice	decreased triglycerides levels cased obesity in blood serum of the host mice	[89]

## Data Availability

No new data were created or analyzed in this study. Data sharing is not applicable to this article.

## References

[1] Yi Y., Li P., Zhao F., Zhang T., Shan Y., Wang X., Liu B., Chen Y., Zhao X., Lü X. (2022). Current status and potentiality of class II bacteriocins from lactic acid bacteria: Structure, mode of action and applications in the food industry. Trends Food Sci. Technol..

[2] Ismael M., Gu Y., Cui Y., Wang T., Yue F., Yantin Q., Lü X. (2022). Lactic acid bacteria isolated from Chinese traditional fermented milk as novel probiotic strains and their potential therapeutic applications. 3 Biotech.

[3] Ismael M., Gu Y., Cui Y., Wang T., Yue F., Qin Y., Lü X. (2022). Probiotic of *Lactiplantibacillus plantarum* NWAFU-BIO-BS29 Isolated from Chinese Traditional Fermented Milk and Its Potential Therapeutic Applications Based on Gut Microbiota Regulation. Foods.

[4] Meade E., Slattery M.A., Garvey M. (2020). *Bacteriocins*, Potent Antimicrobial Peptides and the Fight against Multi Drug Resistant Species: Resistance Is Futile?. Antibiotics.

[5] Fernandes A., Jobby R. (2022). *Bacteriocins* from lactic acid bacteria and their potential clinical applications. Appl. Biochem. Biotechnol..

[6] Liu Q., Yu Z., Tian F., Zhao J., Zhang H., Zhai Q., Chen W. (2020). Surface components and metabolites of probiotics for regulation of intestinal epithelial barrier. Microb. Cell Fact..

[7] Tang H.W., Phapugrangkul P., Fauzi H.M., Tan J.S. (2021). Lactic Acid Bacteria Bacteriocin, an Antimicrobial Peptide Effective Against Multidrug Resistance: A Comprehensive Review. Int. J. Pept. Res. Ther..

[8] Mu Y., Zhang C., Jin C.Z., Li T., Jin F.J., Lee H.G., Jin L. (2024). Antibacterial activity and action mode of crude bacteriocin C2-1 from *Ligilactobacillus salivarius* C2-1 against *Listeria monocytogenes* CICC 21633. LWT.

[9] Molujin A.M., Abbasiliasi S., Nurdin A., Lee P.-C., Gansau J.A., Jawan R. (2022). *Bacteriocins* as Potential Therapeutic Approaches in the Treatment of Various Cancers: A Review of In Vitro Studies. Cancers.

[10] Rani P., Tiwari S.K., Kumar A., Bilal M., Ferreira L.F.R., Kumari M. (2023). Chapter 6—Health benefits of bacteriocins produced by probiotic lactic acid bacteria. Microbial Biomolecules.

[11] Peng Z., Wang D.L., He Y.Y., Wei Z.Q., Xie M.Y., Xiong T. (2024). Gut Distribution, Impact Factor, and Action Mechanism of Bacteriocin-Producing Beneficial Microbes as Promising Antimicrobial Agents in Gastrointestinal Infection. Probiotics Antimicrob. Proteins.

[12] Wang T., Wang S., Dong S., Zhang Y., Ismael M., Wang S., Shi C., Yang J., Wang X., Lü X. (2022). Interaction of *Companilactobacillus crustorum* MN047-derived bacteriocins with gut microbiota. Food Chem..

[13] Huang R., Wu F., Zhou Q., Wei W., Yue J., Xiao B., Luo Z. (2022). Lactobacillus and intestinal diseases: Mechanisms of action and clinical applications. Microbiol. Res..

[14] Bhatia A., Shatanof R.A., Bordoni B. (2023). Embryology, Gastrointestinal. StatPearls.

[15] Ramirez-Olea H., Reyes-Ballesteros B., Chavez-Santoscoy R.A. (2022). Potential application of the probiotic *Bacillus licheniformis* as an adjuvant in the treatment of diseases in humans and animals: A systematic review. Front. Microbiol..

[16] Taherikalani M., Ghafourian S. (2021). Anticancer properties of colicin E7 against colon cancer. Prz. Gastroenterol..

[17] Deng X., Yang J., Zhang Y., Chen X., Wang C., Suo H., Song J. (2023). An Update on the Pivotal Roles of Probiotics, Their Components, and Metabolites in Preventing Colon Cancer. Foods.

[18] Zhang S., Xiao X., Yi Y., Wang X., Zhu L., Shen Y., Lin D., Wu C. (2024). Tumor initiation and early tumorigenesis: Molecular mechanisms and interventional targets. Signal Transduct. Target. Ther..

[19] Ankaiah D., Palanichamy E., Antonyraj C.B., Ayyanna R., Perumal V., Ahamed S.I.B., Arul V. (2018). Cloning, overexpression, purification of bacteriocin enterocin-B and structural analysis, interaction determination of enterocin-A, B against pathogenic bacteria and human cancer cells. Int. J. Biol. Macromol..

[20] Kaur S., Kaur S. (2015). *Bacteriocins* as Potential Anticancer Agents. Front. Pharmacol..

[21] Sexton R.E., Al Hallak M.N., Diab M., Azmi A.S. (2020). Gastric cancer: A comprehensive review of current and future treatment strategies. Cancer Metastasis Rev..

[22] Dikeocha I.J., Al-Kabsi A.M., Ahmeda A.F., Mathai M., Alshawsh M.A. (2023). Investigation into the Potential Role of *Propionibacterium freudenreichii* in Prevention of Colorectal Cancer and Its Effects on the Diversity of Gut Microbiota in Rats. Int. J. Mol. Sci..

[23] Joo N.E., Ritchie K., Kamarajan P., Miao D., Kapila Y.L. (2012). Nisin, an apoptogenic bacteriocin and food preservative, attenuates HNSCC tumorigenesis via CHAC1. Cancer Med..

[24] Goh K.S., Ng Z.J., Halim M., Oslan S.N., Oslan S.N.H., Tan J.S. (2022). A Comprehensive Review on the Anticancer Potential of Bacteriocin: Preclinical and Clinical Studies. Int. J. Pept. Res. Ther..

[25] Fathizadeh H., Saffari M., Esmaeili D., Moniri R., Kafil H.S. (2021). *Bacteriocins*: New Potential Therapeutic Candidates in Cancer Therapy. Curr. Mol. Med..

[26] Bernardes N., Chakrabarty A.M., Fialho A.M. (2013). Engineering of bacterial strains and their products for cancer therapy. Appl. Microbiol. Biotechnol..

[27] Norouzi Z., Salimi A., Halabian R., Fahimi H. (2018). Nisin, a potent bacteriocin and anti-bacterial peptide, attenuates expression of metastatic genes in colorectal cancer cell lines. Microb. Pathog..

[28] Ahmadi S., Ghollasi M., Hosseini H.M. (2017). The apoptotic impact of nisin as a potent bacteriocin on the colon cancer cells. Microb. Pathog..

[29] De Giani A., Bovio F., Forcella M., Fusi P., Sello G., Di Gennaro P. (2019). Identification of a bacteriocin-like compound from *Lactobacillus plantarum* with antimicrobial activity and effects on normal and cancerogenic human intestinal cells. AMB Express.

[30] Villarante K.I., Elegado F.B., Iwatani S., Zendo T., Sonomoto K., de Guzman E.E. (2011). Purification, characterization and in vitro cytotoxicity of the bacteriocin from *Pediococcus acidilactici* K2a2-3 against human colon adenocarcinoma (HT29) and human cervical carcinoma (HeLa) cells. World J. Microbiol. Biotechnol..

[31] Kang S., Guo Y., Rao J., Jin H., You H.J., Ji G.E. (2021). In vitro and in vivo inhibition of *Helicobacter pylori* by *Lactobacillus plantarum* pH3A, monolaurin, and grapefruit seed extract. Food Funct..

[32] Penumetcha S.S., Ahluwalia S., Irfan R., Khan S.A., Rohit Reddy S., Vasquez Lopez M.E., Zahid M., Busmail A., Mohammed L. (2021). The Efficacy of Probiotics in the Management of Helicobacter Pylori: A Systematic Review. Cureus.

[33] Kim T.S., Hur J.W., Yu M.A., Cheigh C.I., Kim K.N., Hwang J.K., Pyun Y.R. (2003). Antagonism of *Helicobacter pylori* by bacteriocins of lactic acid bacteria. J. Food Prot..

[34] De Vuyst L., Vincent P., Makras E., Leroy F., Pot B. (2010). Peptide Extracts from Cultures of Certain Lactobacilli Inhibit *Helicobacter pylori*. Probiotics Antimicrob. Proteins.

[35] Simova E.D., Beshkova D.B., Dimitrov Z.P. (2009). Characterization and antimicrobial spectrum of bacteriocins produced by lactic acid bacteria isolated from traditional Bulgarian dairy products. J. Appl. Microbiol..

[36] Boyanova L., Gergova G., Markovska R., Yordanov D., Mitov I. (2017). Bacteriocin-like inhibitory activities of seven *Lactobacillus delbrueckii* subsp. *bulgaricus* strains against antibiotic susceptible and resistant *Helicobacter pylori* strains. Lett. Appl. Microbiol..

[37] Kingkaew E., Woraprayote W., Booncharoen A., Niwasabutra K., Janyaphisan T., Vilaichone R.-K., Yamaoka Y., Visessanguan W., Tanasupawat S. (2023). Functional genome analysis and anti-*Helicobacter pylori* activity of a novel bacteriocinogenic *Lactococcus* sp. NH2-7C from Thai fermented pork (Nham). Sci. Rep..

[38] Liang Y., Yan J., Chen Z., Gu Q., Li P. (2022). Antibacterial Effects of Bacteriocin PLNC8 against *Helicobacter pylori* and Its Potential Mechanism of Action. Foods.

[39] Lim E.-S. (2015). Purification and characterization of two bacteriocins from *Lactobacillus brevis* BK11 and *Enterococcus faecalis* BK61 showing anti-*Helicobacter pylori* activity. J. Korean Soc. Appl. Biol. Chem..

[40] Krishnamoorthi R., Srinivash M., Mahalingam P.U., Malaikozhundan B., Suganya P., Gurushankar K. (2022). Antimicrobial, anti-biofilm, antioxidant and cytotoxic effects of bacteriocin by *Lactococcus lactis* strain CH3 isolated from fermented dairy products—An in vitro and in silico approach. Int. J. Biol. Macromol..

[41] Haranahalli Nataraj B., Naithani H., Nagpal R., Behare P.V., Singh J., Vyas A. (2022). Chapter 23—*Bacteriocins* and antimicrobial peptides as an alternative to antibiotics. Advances in Dairy Microbial Products.

[42] Escolà-Vergé L., Los-Arcos I., Almirante B. (2020). New antibiotics for the treatment of infections by multidrug-resistant microorganisms. Med. Clínica (Engl. Ed.).

[43] Parmanik A., Das S., Kar B., Bose A., Dwivedi G.R., Pandey M.M. (2022). Current Treatment Strategies Against Multidrug-Resistant Bacteria: A Review. Curr. Microbiol..

[44] Varas M.A., Muñoz-Montecinos C., Kallens V., Simon V., Allende M.L., Marcoleta A.E., Lagos R. (2020). Exploiting Zebrafish Xenografts for Testing the in vivo Antitumorigenic Activity of Microcin E492 Against Human Colorectal Cancer Cells. Front. Microbiol..

[45] Lagos R., Tello M., Mercado G., García V., Monasterio O. (2009). Antibacterial and antitumorigenic properties of microcin E492, a pore-forming bacteriocin. Curr. Pharm. Biotechnol..

[46] Lim S.-M. (2014). Anti-*Helicobacter pylori* activity of antimicrobial substances produced by lactic acid bacteria isolated from Baikkimchi. J. Korean Soc. Appl. Biol. Chem..

[47] Ahn H., Lee G., Lee W., Kim M., Lee K.-G. (2023). Evaluation of probiotic and anti-inflammatory properties of bacteriocinogenic *Pediococcus acidilactici* HW01 and *Leuconostoc citreum* HW02 from malted barley. Chem. Biol. Technol. Agric..

[48] Ismael M., Qayyum N., Gu Y., Zhezhe Y., Cui Y., Zhang Y., Lü X. (2023). Protective effect of plantaricin bio-LP1 bacteriocin on multidrug-resistance Escherichia Coli infection by alleviate the inflammation and modulate of gut-microbiota in BALB/c mice model. Int. J. Biol. Macromol..

[49] Li M., Ge Q., Du H., Jiang P., Bao Z., Chen D., Lin S. (2021). Potential Mechanisms Mediating the Protective Effects of *Tricholoma matsutake*-Derived Peptides in Mitigating DSS-Induced Colitis. J. Agric. Food Chem..

[50] Jabés D., Brunati C., Candiani G., Riva S., Romanó G., Donadio S. (2011). Efficacy of the new lantibiotic NAI-107 in experimental infections induced by multidrug-resistant Gram-positive pathogens. Antimicrob. Agents Chemother..

[51] Kamel B., Hamma-Faradji S., Meddour A., Belguesmia Y., Cudennec B., Bendali F., Daube G., Taminiau B., Drider D. (2021). Gut microbiota, body weight and histopathological examinations in experimental infection by methicillin-resistant *Staphylococcus aureus*: Antibiotic versus bacteriocin. Benef. Microbes.

[52] Yi L., Dang J., Zhang L., Wu Y., Liu B., Lü X. (2016). Purification, characterization and bactericidal mechanism of a broad spectrum bacteriocin with antimicrobial activity against multidrug-resistant strains produced by *Lactobacillus coryniformis* XN8. Food Control.

[53] Baindara P., Chaudhry V., Mittal G., Liao L.M., Matos C.O., Khatri N., Franco O.L., Patil P.B., Korpole S. (2016). Characterization of the Antimicrobial Peptide Penisin, a Class Ia Novel Lantibiotic from *Paenibacillus* sp. Strain A3. Antimicrob. Agents Chemother..

[54] Benítez-Chao D.F., León-Buitimea A., Lerma-Escalera J.A., Morones-Ramírez J.R. (2021). *Bacteriocins*: An Overview of Antimicrobial, Toxicity, and Biosafety Assessment by in vivo Models. Front. Microbiol..

[55] Gu Y., Ismael M., Wang X., Liu B., Shan Y., Chen Y., Zhou Y., Yi Y., Lü X. (2021). Mining and heterologous expression of bacteriocins from *Limosilactobacillus fermentum* LBM97. Food Biosci..

[56] Gonzalez-Ochoa G., Flores-Mendoza L.K., Icedo-Garcia R., Gomez-Flores R., Tamez-Guerra P. (2017). Modulation of rotavirus severe gastroenteritis by the combination of probiotics and prebiotics. Arch. Microbiol..

[57] Harper A., Vijayakumar V., Ouwehand A.C., Ter Haar J., Obis D., Espadaler J., Binda S., Desiraju S., Day R. (2020). Viral Infections, the Microbiome, and Probiotics. Front. Cell Infect. Microbiol..

[58] Umair M., Jabbar S., Zhaoxin L., Jianhao Z., Abid M., Khan K.R., Korma S.A., Alghamdi M.A., El-Saadony M.T., Abd El-Hack M.E. (2022). Probiotic-Based Bacteriocin: Immunity Supplementation Against Viruses. An Updated Review. Front. Microbiol..

[59] Offit P.A. (2018). Challenges to Developing a Rotavirus Vaccine. Viral Immunol..

[60] Tompa D.R., Immanuel A., Srikanth S., Kadhirvel S. (2021). Trends and strategies to combat viral infections: A review on FDA approved antiviral drugs. Int. J. Biol. Macromol..

[61] Wachsman M.B., Castilla V., de Ruiz Holgado A.P., de Torres R.A., Sesma F., Coto C.E. (2003). Enterocin CRL35 inhibits late stages of HSV-1 and HSV-2 replication in vitro. Antivir. Res..

[62] Serkedjieva J., Danova S., Ivanova I. (2000). Antiinfluenza virus activity of a bacteriocin produced by *Lactobacillus delbrueckii*. Appl. Biochem. Biotechnol..

[63] Drider D., Bendali F., Naghmouchi K., Chikindas M.L. (2016). *Bacteriocins*: Not Only Antibacterial Agents. Probiotics Antimicrob. Proteins.

[64] Kuniyoshi T.M., O’Connor P.M., Lawton E., Thapa D., Mesa-Pereira B., Abulu S., Hill C., Ross R.P., Oliveira R.P.S., Cotter P.D. (2022). An oxidation resistant pediocin PA-1 derivative and penocin A display effective anti-Listeria activity in a model human gut environment. Gut Microbes.

[65] Piper C., Casey P.G., Hill C., Cotter P.D., Ross R.P. (2012). The Lantibiotic Lacticin 3147 Prevents Systemic Spread of *Staphylococcus aureus* in a Murine Infection Model. Int. J. Microbiol..

[66] Okuda K., Zendo T., Sugimoto S., Iwase T., Tajima A., Yamada S., Sonomoto K., Mizunoe Y. (2013). Effects of bacteriocins on methicillin-resistant *Staphylococcus aureus* biofilm. Antimicrob. Agents Chemother..

[67] Kim J.H., Ahn H., Lee D., Lee H., Kim W.J. (2023). Antibiofilm activity of crude bacteriocin JM01 produced by *Pediococcus acidilactici* against methicillin-resistant *Staphylococcus aureus* (MRSA). Int. J. Food Sci. Technol..

[68] Yi E.-J., Kim A.-J. (2023). Antimicrobial and Antibiofilm Effect of Bacteriocin-Producing Pediococcus inopinatus K35 Isolated from Kimchi against Multidrug-Resistant *Pseudomonas aeruginosa*. Antibiotics.

[69] Kruszewska D., Sahl H.G., Bierbaum G., Pag U., Hynes S.O., Ljungh A. (2004). Mersacidin eradicates methicillin-resistant *Staphylococcus aureus* (MRSA) in a mouse rhinitis model. J. Antimicrob. Chemother..

[70] Anjana, Tiwari S.K. (2022). Bacteriocin-Producing Probiotic Lactic Acid Bacteria in Controlling Dysbiosis of the Gut Microbiota. Front. Cell. Infect. Microbiol..

[71] Abdi M., Lohrasbi V., Asadi A., Esghaei M., Jazi F.M., Rohani M., Talebi M. (2021). Interesting probiotic traits of mother’s milk *Lactobacillus isolates*; from bacteriocin to inflammatory bowel disease improvement. Microb. Pathog..

[72] Torres N.I., Noll K.S., Xu S., Li J., Huang Q., Sinko P.J., Wachsman M.B., Chikindas M.L. (2013). Safety, formulation, and in vitro antiviral activity of the antimicrobial peptide subtilosin against herpes simplex virus type 1. Probiotics Antimicrob. Proteins.

[73] Soltani S., Hammami R., Cotter P.D., Rebuffat S., Said L.B., Gaudreau H., Bédard F., Biron E., Drider D., Fliss I. (2021). *Bacteriocins* as a new generation of antimicrobials: Toxicity aspects and regulations. FEMS Microbiol. Rev..

[74] Cavicchioli V.Q., Carvalho O.V.d., Paiva J.C.d., Todorov S.D., Silva Júnior A., Nero L.A. (2018). Inhibition of herpes simplex virus 1 (HSV-1) and poliovirus (PV-1) by bacteriocins from *Lactococcus lactis* subsp. *lactis* and *Enterococcus durans* strains isolated from goat milk. Int. J. Antimicrob. Agents.

[75] Al Kassaa I., Hober D., Hamze M., Chihib N.E., Drider D. (2014). Antiviral Potential of Lactic Acid Bacteria and Their *Bacteriocins*. Probiotics Antimicrob. Proteins.

[76] Férir G., Petrova M.I., Andrei G., Huskens D., Hoorelbeke B., Snoeck R., Vanderleyden J., Balzarini J., Bartoschek S., Brönstrup M. (2013). The lantibiotic peptide labyrinthopeptin A1 demonstrates broad anti-HIV and anti-HSV activity with potential for microbicidal applications. PLoS ONE.

[77] Yin X., Heeney D., Srisengfa Y., Golomb B., Griffey S., Marco M. (2017). Bacteriocin biosynthesis contributes to the anti-inflammatory capacities of probiotic *Lactobacillus plantarum*. Benef. Microbes.

[78] Butorac K., Novak J., Banić M., Leboš Pavunc A., Čuljak N., Oršolić N., Odeh D., Perica J., Šušković J., Kos B. (2023). Modulation of the Gut Microbiota by the Plantaricin-Producing *Lactiplantibacillus plantarum* D13, Analysed in the DSS-Induced Colitis Mouse Model. Int. J. Mol. Sci..

[79] Lay C.L., Dridi L., Bergeron M.G., Ouellette M., Fliss I.l. (2016). Nisin is an effective inhibitor of *Clostridium difficile* vegetative cells and spore germination. J. Med Microbiol..

[80] Majeed H., Gillor O., Kerr B., Riley M.A. (2011). Competitive interactions in *Escherichia coli* populations: The role of bacteriocins. ISME J..

[81] Hols P., Ledesma-García L., Gabant P., Mignolet J. (2019). Mobilization of Microbiota Commensals and Their *Bacteriocins* for Therapeutics. Trends Microbiol..

[82] Hurt R.T., Kulisek C., Buchanan L.A., McClave S.A. (2010). The obesity epidemic: Challenges, health initiatives, and implications for gastroenterologists. Gastroenterol. Hepatol..

[83] Bai L., Kumar S., Verma S., Seshadri S. (2020). Bacteriocin PJ4 from probiotic lactobacillus reduced adipokine and inflammasome in high fat diet induced obesity. 3 Biotech.

[84] Ruban A., Stoenchev K., Ashrafian H., Teare J. (2019). Current treatments for obesity. Clin. Med..

[85] Al-Emarah M.K., Kazerani H.R., Taghizad F., Dehghani H., Elahi M. (2023). Anti-obesity effect of the bacterial product nisin in an NIH Swiss mouse model. Lipids Health Dis..

[86] Heeney D.D., Zhai Z., Bendiks Z., Barouei J., Martinic A., Slupsky C., Marco M.L. (2019). *Lactobacillus plantarum* bacteriocin is associated with intestinal and systemic improvements in diet-induced obese mice and maintains epithelial barrier integrity in vitro. Gut Microbes.

[87] Taghizad F., Kazerani H.R., Dehghani H., Asoodeh A., Yaghubi D. (2021). A novel approach towards obesity: The use of a bacterial product, gassericin A, in 3T3-L1 cells. Obes. Res. Clin. Pract..

[88] Kumari R., Singhvi N., Sharma P., Choudhury C., Shakya R. (2023). Virtual screening of gut microbiome bacteriocins as potential inhibitors of stearoyl-CoA desaturase 1 to regulate adipocyte differentiation and thermogenesis to combat obesity. J. Biomol. Struct. Dyn..

[89] Umu Ö.C., Bäuerl C., Oostindjer M., Pope P.B., Hernández P.E., Pérez-Martínez G., Diep D.B. (2016). The Potential of Class II *Bacteriocins* to Modify Gut Microbiota to Improve Host Health. PLoS ONE.

[90] Teng K., Huang F., Liu Y., Wang Y., Xia T., Yun F., Zhong J. (2023). Food and gut originated bacteriocins involved in gut microbe-host interactions. Crit. Rev. Microbiol..

[91] Ma T., Shen X., Shi X., Sakandar H.A., Quan K., Li Y., Jin H., Kwok L.-Y., Zhang H., Sun Z. (2023). Targeting gut microbiota and metabolism as the major probiotic mechanism—An evidence-based review. Trends Food Sci. Technol..

[92] Bu Y., Liu Y., Zhang T., Liu Y., Zhang Z., Yi H. (2023). Bacteriocin-Producing *Lactiplantibacillus plantarum* YRL45 Enhances Intestinal Immunity and Regulates Gut Microbiota in Mice. Nutrients.

[93] Qiao Y., Qiu Z., Tian F., Yu L., Zhao J., Zhang H., Zhai Q., Chen W. (2022). Effect of bacteriocin-producing *Pediococcus acidilactici* strains on the immune system and intestinal flora of normal mice. Food Sci. Hum. Wellness.

[94] Dimidi E., Cox S.R., Rossi M., Whelan K. (2019). Fermented Foods: Definitions and Characteristics, Impact on the Gut Microbiota and Effects on Gastrointestinal Health and Disease. Nutrients.

[95] Patzer S.I., Baquero M.R., Bravo D., Moreno F., Hantke K. (2003). The colicin G, H and X determinants encode microcins M and H47, which might utilize the catecholate siderophore receptors FepA, Cir, Fiu and IroN. Microbiology.

[96] van Staden D.A., Brand A.M., Endo A., Dicks L.M. (2011). Nisin F, intraperitoneally injected, may have a stabilizing effect on the bacterial population in the gastro-intestinal tract, as determined in a preliminary study with mice as model. Lett. Appl. Microbiol..

[97] Donia M.S., Fischbach M.A. (2015). Small molecules from the human microbiota. Science.

[98] Cox C.R., Coburn P.S., Gilmore M.S. (2005). Enterococcal cytolysin: A novel two component peptide system that serves as a bacterial defense against eukaryotic and prokaryotic cells. Curr. Protein Pept. Sci..

[99] Mann E.R., Lam Y.K., Uhlig H.H. (2024). Short-chain fatty acids: Linking diet, the microbiome and immunity. Nat. Rev. Immunol..

[100] Pu J., Hang S., Liu M., Chen Z., Xiong J., Li Y., Wu H., Zhao X., Liu S., Gu Q. (2022). A Class IIb Bacteriocin Plantaricin NC8 Modulates Gut Microbiota of Different Enterotypes in vitro. Front. Nutr..

[101] Peterson L.W., Artis D. (2014). Intestinal epithelial cells: Regulators of barrier function and immune homeostasis. Nat. Rev. Immunol..

[102] Barbara G., Barbaro M.R., Fuschi D., Palombo M., Falangone F., Cremon C., Marasco G., Stanghellini V. (2021). Inflammatory and Microbiota-Related Regulation of the Intestinal Epithelial Barrier. Front. Nutr..

[103] Gu Q., Yan J., Lou Y., Zhang Z., Li Y., Zhu Z., Liu M., Wu D., Liang Y., Pu J. (2024). *Bacteriocins*: Curial guardians of gastrointestinal tract. Compr. Rev. Food Sci. Food Saf..

[104] Ni Z.J., Zhang X.Y., Liu F., Wang M., Hao R.H., Ling P.X., Zhu X.Q. (2017). Effect of Co-overexpression of Nisin Key Genes on Nisin Production Improvement in *Lactococcus lactis* LS01. Probiotics Antimicrob. Proteins.

[105] Huang F., Teng K., Liu Y., Cao Y., Wang T., Ma C., Zhang J., Zhong J. (2021). *Bacteriocins*: Potential for Human Health. Oxid. Med. Cell Longev..

[106] Daba G.M., Elkhateeb W.A. (2020). *Bacteriocins* of lactic acid bacteria as biotechnological tools in food and pharmaceuticals: Current applications and future prospects. Biocatal. Agric. Biotechnol..

[107] Maurya A.P., Maurya V.K., Thakur R.L., Egbuna C., Mishra A.P., Goyal M.R. (2021). Chapter 16—Bacteriocin producing lactic acid bacteria: Their relevance to human nutrition and health. Preparation of Phytopharmaceuticals for the Management of Disorders.

[108] Woo C.W., Tso P., Yiu J.H.C. (2022). Commensal gut microbiota-based strategies for oral delivery of therapeutic proteins. Trends Pharmacol. Sci..

[109] Dalton J., Dalton J. (2019). SWOT Analysis (Strengths, Weaknesses, Opportunities, Threats). Great Big Agile: An OS for Agile Leaders.

[110] Mukherjee A., Breselge S., Dimidi E., Marco M.L., Cotter P.D. (2023). Fermented foods and gastrointestinal health: Underlying mechanisms. Nat. Rev. Gastroenterol. Hepatol..

[111] Cui Y., Luo L., Wang X., Lu Y., Yi Y., Shan Y., Liu B., Zhou Y., Lü X. (2021). Mining, heterologous expression, purification, antibactericidal mechanism, and application of bacteriocins: A review. Compr. Rev. Food Sci. Food Saf..

